# Serum Uric Acid Levels in the First Trimester of Pregnancy and the Development of Gestational Diabetes Mellitus: A Prospective Cohort Study

**DOI:** 10.7759/cureus.115200

**Published:** 2026-08-25

**Authors:** Parth Dhande, Kanchan S Dwidmuthe, Anuja Bhalerao

**Affiliations:** 1 Obstetrics and Gynaecology, NKP Salve Institute of Medical Sciences and Research Centre and Lata Mangeshkar Hospital, Nagpur, IND; 2 Obstetrics and Gynaecology, NKP Salve Institute of Medical Sciences and Research Centre, Nagpur, IND

**Keywords:** dipsi, gestational diabetes mellitus, pgbs, screeening, serum uric acid level

## Abstract

Introduction: Gestational diabetes mellitus (GDM) is a common metabolic disorder of pregnancy associated with adverse maternal and fetal outcomes; early identification of at-risk women is essential for timely intervention. Serum uric acid (SUA) has emerged as a potential biomarker for predicting glucose intolerance during pregnancy. This study aimed to evaluate the association between first-trimester SUA levels and the subsequent development of GDM.

Materials and methods: A prospective cohort study was conducted in the Department of Obstetrics and Gynaecology of a tertiary care teaching hospital from January 2024 to December 2025. A total of 212 non-diabetic antenatal women in their first trimester were enrolled using consecutive sampling. Baseline clinical assessment, anthropometric measurements, and SUA estimation were performed at enrolment using a fully automated biochemical analyzer (Dimension RXL Max, Siemens Healthineers, Erlangen, Germany). Participants were followed until 24-28 weeks of gestation and screened for GDM using the Diabetes in Pregnancy Study Group of India (DIPSI) criteria following a 75 g oral glucose challenge. Statistical analysis was performed using SPSS v25.0 (IBM Corp., Armonk, NY). Independent t-test, chi-square test, Pearson correlation, multivariate logistic regression, and receiver operating characteristic (ROC) curve analyses were used.

Results: Of the 212 participants, 44 (20.8%) developed GDM. Women who developed GDM had significantly higher first-trimester SUA levels (5.4 ± 0.9 mg/dL vs. 3.8 ± 0.8 mg/dL; p < 0.001). SUA showed a positive correlation with second-trimester post-glucose blood sugar (r = 0.52, p < 0.001). The unadjusted odds ratio for GDM with SUA >4 mg/dL was 12.0 (95% CI: 4.8-30.0; p < 0.001). On multivariate logistic regression, elevated SUA (adjusted odds ratio [AOR] = 3.6, 95% CI: 1.8-7.1, p < 0.001), BMI ≥25 kg/m² (AOR = 2.9), and positive family history of diabetes (AOR = 2.4) were independent predictors. ROC analysis demonstrated an area under the curve (AUC) of 0.87 at a cut-off of 4.1 mg/dL (sensitivity 80%, specificity 70%).

Conclusions: Elevated first-trimester SUA levels are significantly associated with, and independently predict, GDM. SUA may serve as a simple, inexpensive, and widely available biomarker for early identification of women at increased risk of GDM. Large-scale multicenter studies are warranted to validate these findings and establish ethnicity-specific thresholds.

## Introduction

Gestational diabetes mellitus (GDM) refers to carbohydrate intolerance of any severity that begins, or is first identified, during pregnancy. When unrecognised or poorly controlled, it raises the likelihood of several adverse obstetric outcomes, including excessive fetal growth, shoulder dystocia, birth trauma, preterm delivery, perinatal death, and the need for caesarean delivery [1]. Its impact is not confined to the index pregnancy: women who develop GDM subsequently face a markedly higher lifetime risk of type 2 diabetes mellitus (T2DM) and cardiovascular disease [2].

GDM ranks among the most common complications encountered in pregnancy worldwide. The International Diabetes Federation estimates that hyperglycaemia complicates roughly 14% of pregnancies globally, with GDM accounting for most of these cases [3]. Reported prevalence varies considerably by region: in the United States, diabetes affects approximately 7% of pregnancies, of which 86% is gestational [4], while pooled European estimates place prevalence at 10.9% [5]. India shows an especially broad range, from 3% to 35% depending on the population and diagnostic criteria applied, with Indian women reported to carry up to an 11-fold higher risk relative to women from certain other regions [6,7].

Normal pregnancy is marked by a progressive rise in insulin resistance, driven largely by counter-regulatory placental hormones such as human placental lactogen, progesterone, oestrogen, cortisol, and placental growth hormone. GDM develops when maternal pancreatic ß-cells fail to secrete sufficient insulin to compensate for this heightened resistance, resulting in hyperglycaemia [8]. Because routine screening for GDM is performed only at 24-28 weeks of gestation, women who are already at elevated risk may go unidentified until relatively late in pregnancy, limiting the window for timely preventive intervention.

Serum uric acid (SUA), the end product of purine catabolism, has attracted interest as a candidate early biomarker for GDM because of its established roles in insulin resistance and low-grade inflammation-both central to GDM pathogenesis [9]. Under normal physiological conditions, SUA declines in early pregnancy as oestrogen promotes uricosuria and glomerular filtration rises; an SUA level that remains elevated or rises during the first trimester may therefore reflect an underlying metabolic vulnerability that predates conception [10]. Several studies have reported an association between elevated first-trimester SUA and subsequent GDM, although the strength of this association has varied across populations [11].

Because SUA measurement is inexpensive, widely available, and technically simple, first-trimester SUA estimation could, if validated, serve as a practical addition to existing antenatal screening pathways. We therefore undertook this prospective cohort study to examine the association between first-trimester SUA levels and subsequent GDM development, with the aim of assessing its potential value as an early predictive biomarker.

## Materials and methods

Study design and setting

This was a prospective cohort study conducted in the outpatient department (OPD) and antenatal care (ANC) ward of the Department of Obstetrics and Gynaecology at a tertiary care teaching hospital over a 24-month period from January 2024 to December 2025.

Participants

All antenatal women in the first trimester of pregnancy attending for routine prenatal examination during the study period were screened for eligibility. Women who fulfilled the inclusion criteria and provided written informed consent were enrolled consecutively.

Inclusion and Exclusion Criteria

The inclusion criteria comprised non-diabetic antenatal women in the first trimester of pregnancy (up to 13 completed weeks of gestation). Participants with known diabetes mellitus, hypertension, gout, multiple pregnancies, smoking, use of drugs known to affect SUA levels (aspirin, phenothiazines, diuretics, steroids), liver disease, or chronic kidney disease were excluded.

Ethical considerations

This study was approved by the Institutional Ethics Committee of NKP Salve Institute of Medical Sciences and Research Centre and Lata Mangeshkar Hospital Nagpur (approval number: 47/2024), and was conducted in accordance with the Declaration of Helsinki. Written informed consent was obtained from all participants prior to enrolment, and patient confidentiality was maintained throughout the study.

Sample size

The minimum required sample size was calculated using the formula n = Z²p(1−p)/d², where Z = 1.96 (95% confidence level), p = 0.1655 (prevalence of GDM in India; Seshiah et al., 2004 [12]), and d = 0.05 (margin of error). This yielded a calculated minimum of 212.3, rounded up to 213 participants. During the 24-month study period, 212 eligible women were enrolled consecutively and followed prospectively to GDM ascertainment at 24-28 weeks of gestation; all enrolled participants completed follow-up, with no losses to follow-up or missing outcome data. The achieved sample of 212 therefore closely approximates the calculated minimum requirement, and no separate inflation for anticipated attrition was applied, as none of the enrolled participants were lost to follow-up during the observation period.

Data collection and procedures

At enrolment, a pre-designed, pre-validated proforma was used to record detailed history including obstetric, medical, surgical, family, and personal history. A thorough general physical examination was performed, including anthropometric measurements (height, weight, and body mass index [BMI] calculated as weight in kilograms divided by the square of height in metres). Vital signs were recorded, and systemic and obstetric examinations were completed.

For SUA estimation, approximately 2 mL of venous blood was collected in a clot activator tube under aseptic precautions. SUA was measured using a fully automated biochemical analyser (Dimension RXL Max, Siemens Healthineers, Erlangen, Germany) with URCA Flex reagent cartridges and CHEM 1 calibrator, within 3-5 days of collection; samples were stored at 2-8°C.

Screening for GDM at 24-28 weeks was performed using the single-step Diabetes in Pregnancy Study Group of India (DIPSI) glucose challenge test. A 75 g oral glucose load in 300 mL water was administered irrespective of fasting status. Venous blood was collected 2 hours later in a fluoride bulb, and plasma glucose was measured using the Siemens EXL 200 Integrated System (Siemens Healthineers, Erlangen, Germany). A 2-hour plasma glucose value ≥140 mg/dL was diagnostic of GDM per DIPSI criteria [12].

Statistical analysis

Data were analyzed using the Statistical Package for the Social Sciences (SPSS) software, version 25.0 (IBM Corp., Armonk, NY). Continuous variables were expressed as mean ± standard deviation (SD) and were compared using the independent samples t-test. Categorical variables were expressed as frequencies and percentages and were compared using Pearson's chi-square test; all contingency tables had expected cell counts ≥5, satisfying the assumption underlying the chi-square approximation. Pearson correlation coefficients were calculated to assess associations between continuous variables. Logistic regression analysis was performed to identify predictors of GDM, with results expressed as unadjusted and adjusted odds ratios (OR and AOR) with 95% confidence intervals (CI). Multivariate logistic regression included variables significant at p < 0.05 on bivariate analysis. Receiver operating characteristic (ROC) curve analysis was performed to assess the predictive performance of SUA, with the optimal cut-off determined by the Youden index. A p-value < 0.05 was considered statistically significant.

Assessment of Statistical Assumptions

Normality of the continuous variables (age, BMI, and first-trimester SUA) was assessed using the Shapiro-Wilk test together with visual inspection of histograms and Q-Q plots. Age approximated a normal distribution (Shapiro-Wilk p = 0.047; skewness = -0.02), whereas BMI and SUA showed mild-to-moderate positive skew (skewness = 0.56 and 0.69, respectively; Shapiro-Wilk p < 0.001 for both), a pattern commonly observed for clinical variables with a physiological lower bound. Given the adequate group sizes (n = 44 and n = 168) and the modest degree of skew, the independent-samples t-test was considered robust to this deviation under the central limit theorem. Levene's test confirmed homogeneity of variance for all three variables (p > 0.05), supporting the use of the pooled-variance (Student's) t-test rather than the Welch-corrected alternative. As a sensitivity analysis, group comparisons were repeated using the non-parametric Mann-Whitney U test; results were concordant with the t-test for age, BMI, and SUA (all p < 0.05), supporting the robustness of the parametric approach adopted.

For the multivariate logistic regression model, standard model assumptions were verified before interpretation. The events-per-variable ratio (44 GDM events for four covariates, approximately 11:1) met conventional thresholds for stable coefficient estimation. Because all covariates were entered as binary (dichotomised) variables, the linearity-of-the-logit assumption applicable to continuous predictors was not relevant to this model. Multicollinearity among covariates was assessed using variance inflation factors, all of which were below 2.5, indicating no meaningful collinearity. Model calibration was evaluated using the Hosmer-Lemeshow goodness-of-fit test, which was non-significant (χ²(6) = 4.91, p = 0.556), indicating adequate agreement between observed and model-predicted probabilities. Observations were independent by study design, with a single outcome recorded per participant.

## Results

Baseline demographic characteristics

Of the 212 women enrolled, 44 (20.8%) developed GDM and 168 (79.2%) remained normoglycemic. Women in the GDM group were significantly older (28.9 ± 4.2 years vs. 26.8 ± 3.9 years; independent-samples t(210) = 3.13, p = 0.003) and had significantly higher BMI (26.8 ± 3.1 kg/m² vs. 23.9 ± 2.8 kg/m²; t(210) = 5.98, p = 0.001). Gravida status was comparable between groups (χ²(1) = 1.21, p = 0.298) (Table 1).

**Table 1:** Baseline demographic characteristics. *Statistically significant (p < 0.05). GDM, gestational diabetes mellitus; BMI, body mass index

Parameter	GDM (n = 44)	Non-GDM (n = 168)	Test statistic (df), p-value
Mean age (years)	28.9 ± 4.2	26.8 ± 3.9	t(210) = 3.13; p = 0.003*
BMI (kg/m²)	26.8 ± 3.1	23.9 ± 2.8	t(210) = 5.98; p = 0.001*
Primigravida, n (%)	20 (45.5%)	92 (54.8%)	χ²(1) = 1.21; p = 0.298
Multigravida, n (%)	24 (54.5%)	76 (45.2%)	

Area of residence and family history

Most women in both groups resided in rural areas. Area of residence was not significantly associated with GDM development (rural: 63.6% GDM vs. 54.8% non-GDM; χ²(1) = 1.12, p = 0.294). A positive family history of diabetes was significantly more prevalent among women with GDM (50.0% vs. 22.6%; χ²(1) = 12.88, p < 0.001). On multivariate logistic regression, elevated SUA >4.0 mg/dL was the strongest independent predictor of GDM (AOR = 3.6; 95% CI: 1.8-7.1; Wald χ²(1) = 13.39; p < 0.001), followed by BMI ≥25 kg/m² (AOR = 2.9; Wald χ²(1) = 10.04; p = 0.002) and positive family history of diabetes (AOR = 2.4; Wald χ²(1) = 7.64; p = 0.005). Gravida status was not a significant independent predictor (AOR = 1.3; Wald χ²(1) = 0.70; p = 0.312) (Tables 2-3).

**Table 2:** Distribution of area of residence GDM, gestational diabetes mellitus

Area of residence	GDM (n = 44)	Non-GDM (n = 168)	Test statistic (df), p-value
Rural	28 (63.6%)	92 (54.8%)	χ²(1) = 1.12; p = 0.294
Urban	16 (36.4%)	76 (45.2%)	
Total	44 (100%)	168 (100%)	

**Table 3:** Family history of diabetes. *Statistically significant (p < 0.05). GDM, gestational diabetes mellitus

Family history of diabetes	GDM (n = 44)	Non-GDM (n = 168)	Test statistic (df), p-value
Present	22 (50.0%)	38 (22.6%)	χ²(1) = 12.88; p < 0.001*
Absent	22 (50.0%)	130 (77.4%)	
Total	44 (100%)	168 (100%)	

First-trimester SUA levels

Women who developed GDM had markedly higher mean first-trimester SUA (5.4 ± 0.9 mg/dL vs. 3.8 ± 0.8 mg/dL; t(210) = 11.50, p < 0.001) (Table 4).

**Table 4:** First-trimester SUA levels compared between GDM and non-GDM groups. SUA, serum uric acid; SD, standard deviation; GDM, gestational diabetes mellitus

Group	Mean SUA ± SD (mg/dL)	Test statistic (df), p-value
GDM (n = 44)	5.4 ± 0.9	t(210) = 11.50; p < 0.001*
Non-GDM (n = 168)	3.8 ± 0.8	

Correlation of SUA with glycemic parameters

First-trimester SUA levels showed a statistically significant moderate positive correlation with second-trimester post-glucose blood sugar (PGBS) values (Pearson r = 0.52, t(210) = 8.82, p < 0.001), indicating that higher first-trimester SUA is associated with higher subsequent glycemic values (Table 5).

**Table 5:** Correlation of first-trimester SUA with second-trimester PGBS SUA, serum uric acid; PGBS, post-glucose blood sugar

Parameter	Correlation coefficient (r)	Test statistic (df), p-value
PGBS	0.52	t(210) = 8.82; p < 0.001*

Association between SUA and GDM incidence

Among women with SUA ≤4.0 mg/dL, only 6 of 116 (5.2%) developed GDM. In contrast, among those with SUA >4.0 mg/dL, 38 of 96 (39.6%) developed GDM (χ²(1) = 37.82, p < 0.001) (Table 6).

**Table 6:** Association between first-trimester SUA and GDM incidence. Chi-square test; *p < 0.001. SUA, serum uric acid; GDM, gestational diabetes mellitus

SUA	GDM (n = 44)	Non-GDM (n = 168)	Total	Test statistic (df), p-value
≤4.0 mg/dL	6 (13.6%)	110 (65.5%)	116	χ²(1) = 37.82; p < 0.001*
>4.0 mg/dL	38 (86.4%)	58 (34.5%)	96	

 Unadjusted odds ratio for GDM

Women with SUA >4.0 mg/dL had approximately 12 times higher odds of developing GDM compared to those with SUA ≤4.0 mg/dL (OR = 12.0, 95% CI: 4.8-30.0; Wald χ²(1) = 28.25, p < 0.001) (Table 7).

**Table 7:** Unadjusted odds ratio for GDM associated with elevated SUA. SUA, serum uric acid; GDM, gestational diabetes mellitus; CI, confidence interval.

Variable	Odds ratio (OR)	95% CI	Wald χ² (df), p-value
SUA >4.0 mg/dL	12	4.8–30.0	28.25 (1); p < 0.001*

Association of BMI with GDM

Among women with BMI ≥25 kg/m², 32 of 90 (35.6%) developed GDM compared with 12 of 122 (9.8%) women with BMI <25 kg/m² (χ²(1) = 20.83, p = 0.001), demonstrating BMI as an important predictor of GDM (Table 8).

**Table 8:** Association of BMI with GDM. *Statistically significant (p < 0.05). GDM, gestational diabetes mellitus; BMI, body mass index

BMI category	GDM (n = 44)	Non-GDM (n = 168)	Test statistic (df), p-value
<25 kg/m²	12 (27.3%)	110 (65.5%)	χ²(1) = 20.83; p = 0.001*
≥25 kg/m²	32 (72.7%)	58 (34.5%)	

Diagnostic accuracy of SUA: ROC analysis

ROC curve analysis identified a SUA cut-off of 4.1 mg/dL as optimal for predicting GDM, with 80% sensitivity, 70% specificity, and an AUC of 0.87 (95% CI: 0.80-0.94), indicating good discriminative accuracy (Table 9).

**Table 9:** Diagnostic performance of first-trimester SUA for predicting GDM (ROC analysis). AUC, area under the curve; ROC, receiver operating characteristic; GDM, gestational diabetes mellitus

Parameter	Value
AUC	0.87
95% CI (AUC)	0.80–0.94
Optimal cut-off	4.1 mg/dL
Sensitivity	80%
Specificity	70%

Multivariate logistic regression

On multivariate logistic regression, elevated SUA >4.0 mg/dL was the strongest independent predictor of GDM (AOR = 3.6; 95% CI: 1.8-7.1; Wald χ²(1) = 13.39; p < 0.001), followed by BMI ≥25 kg/m² (AOR = 2.9; Wald χ²(1) = 10.04; p = 0.002) and positive family history of diabetes (AOR = 2.4; Wald χ²(1) = 7.64; p = 0.005). Gravida status was not a significant independent predictor (AOR = 1.3; Wald χ²(1) = 0.70; p = 0.312) (Table 10).

**Table 10:** Multivariate logistic regression analysis for independent predictors of GDM. AOR, adjusted odds ratio; CI, confidence interval; BMI, body mass index; GDM, gestational diabetes mellitus. *Statistically significant

Variable	AOR	95% CI	Wald χ² (df), p-value
Serum uric acid >4.0 mg/dL	3.6	1.8–7.1	13.39 (1); p < 0.001*
BMI ≥25 kg/m²	2.9	1.5–5.6	10.04 (1); p = 0.002*
Family history of diabetes	2.4	1.3–4.5	7.64 (1); p = 0.005*
Multigravida	1.3	0.7–2.4	0.70 (1); p = 0.312

## Discussion

This prospective cohort study examined whether first-trimester SUA levels are associated with subsequent GDM development among 212 antenatal women. Five findings stood out: GDM developed in 20.8% of the cohort; women who developed GDM had markedly higher first-trimester SUA than those who did not (5.4 ± 0.9 vs. 3.8 ± 0.8 mg/dL; p < 0.001); a threshold of 4.1 mg/dL discriminated GDM with 80% sensitivity, 70% specificity, and an AUC of 0.87; first-trimester SUA correlated positively with second-trimester PGBS (r = 0.52, p < 0.001); and SUA emerged as the strongest independent predictor of GDM on multivariate analysis (AOR = 3.6; 95% CI: 1.8-7.1).

Incidence of GDM

The 20.8% GDM incidence observed here falls within the range reported from other Indian tertiary care centres. Using International Association of Diabetes and Pregnancy Study Groups (IADPSG) criteria, Asati et al. (2025) [13] reported a prevalence of 19.0% in North India, and Seshiah et al. (2008) [14] documented rates between 9.9% and 17.8% across Tamil Nadu. Globally, reported GDM prevalence spans a wide 1-28%, reflecting differences in diagnostic criteria and population characteristics across studies [15].

Demographic risk factors

Consistent with prior literature, women who developed GDM in our cohort were older (t(210) = 3.13, p = 0.003) and had higher BMI (t(210) = 5.98, p = 0.001) than those who did not. Advancing maternal age is thought to reduce pancreatic ß-cell reserve while amplifying hepatic insulin resistance, effectively lowering the glycaemic threshold at which GDM manifests. Family history of diabetes was also more common among GDM cases (50.0% vs. 22.6%; χ²(1) = 12.88, p < 0.001; AOR = 2.4), in keeping with the recognised genetic contribution to GDM risk. Neither gravida status nor area of residence remained independently significant, a pattern consistent with reports that parity loses predictive value once metabolic confounders are accounted for [14].

SUA and GDM: concordance with published literature

The strong, independent SUA-GDM association identified here adds to a rapidly growing evidence base. In a cohort of 1,570 pregnant women, Laughon et al. (2009) [16] found that first-trimester SUA in the top quartile carried a 3.25-fold higher risk of GDM. Li et al. (2024) [17], studying 6,000 participants, similarly described a dose-response relationship between SUA quartile and GDM incidence, and Zhao et al. (2022) [18], in a much larger cohort of 85,609 pregnant women, likewise reported a significant association between elevated first-trimester SUA and GDM. Pooled data point in the same direction: a dose-response meta-analysis by Nikparast et al. (2023) [19] spanning 105,380 participants estimated an adjusted OR of approximately 2.58 (95% CI: 1.89-3.52), and a later meta-analysis of 25,212 GDM cases found SUA levels roughly 44.3 µmol/L higher among women with GDM, with a pooled adjusted OR of 2.35 [20]. Our unadjusted OR of 12.0, derived from a single-centre 2×2 comparison, exceeds these pooled meta-analytic estimates, most plausibly because of our smaller sample, single-centre design, and population-specific characteristics; notably, our adjusted OR of 3.6 moves considerably closer to these published figures once BMI and family history are accounted for.

Our ROC findings were broadly, though not exactly, in line with other cohorts. Şahin Aker et al. (2016) [21], studying 626 women retrospectively, reported a higher AUC of 0.92, with a lower threshold of 3.95 mg/dL achieving 100% sensitivity and 60% specificity. The modestly lower AUC observed in our cohort (0.87, 95% CI: 0.80-0.94, at a 4.1 mg/dL threshold) may reflect differences in ethnic background, dietary purine intake, or the specific GDM diagnostic criteria applied across studies.

Pathophysiological mechanisms

Several converging mechanisms plausibly link elevated first-trimester SUA to subsequent GDM. Uric acid impairs endothelial nitric oxide synthesis, thereby promoting peripheral insulin resistance, and interferes with insulin-mediated glucose uptake in skeletal muscle and adipose tissue; it further activates the NLRP3 inflammasome, driving interleukin-1ß production that can damage pancreatic ß-cells [20]. Xanthine oxidase-derived reactive oxygen species generated during uric acid metabolism additionally promote hepatic gluconeogenesis. Together, these pathways offer a biologically coherent explanation for why an atypical rise in first-trimester SUA might both predict and actively contribute to the insulin resistance underlying GDM.

Clinical implications

As a first-trimester biomarker, SUA has several practical advantages: it does not require fasting or a glucose load, can be measured using inexpensive, standardized assays, and is readily available even in resource-constrained settings. The multivariate model presented here suggests that a composite risk score combining SUA, BMI, and family history could hold clinical value and merits prospective validation. It is also plausible that dietary counselling targeted at women with elevated first-trimester SUA--for instance, reducing intake of fructose and purine-rich foods--could help lower GDM risk in this subgroup, though this remains to be tested directly.

Limitations

Several limitations warrant consideration. Because this was a single-centre study conducted in a tertiary care setting, the findings may not generalise fully to community-based populations. We did not systematically capture dietary purine intake, hydration status, or physical activity, all of which can influence SUA levels, nor did we measure insulin resistance indices such as HOMA-IR or inflammatory markers. SUA was measured only once, in the first trimester; repeated measurements across pregnancy would have offered insight into its temporal trajectory. Larger, multicentre prospective studies incorporating standardised dietary assessment and serial SUA measurement are needed to isolate the independent contribution of hyperuricaemia from related metabolic factors.

## Conclusions

In this cohort, elevated first-trimester SUA was significantly associated with subsequent GDM and remained the strongest independent predictor after adjusting for BMI and family history. A threshold of 4.1 mg/dL provided good discriminative accuracy (AUC = 0.87, 95% CI: 0.80-0.94). Given that SUA testing is inexpensive, requires no fasting, and is widely accessible, it may have a role in first-trimester antenatal risk stratification for GDM, potentially enabling earlier preventive intervention. Before routine clinical adoption, however, these findings require confirmation in larger, multicentre studies that establish standardised, population-specific SUA thresholds.
